# Skeletal age prediction models by maturity status in male soccer players

**DOI:** 10.1038/s41598-025-00402-x

**Published:** 2025-05-25

**Authors:** Luis Alberto Flores, Christopher McLaren-Towlson, Lidia G. De León, Fabiana Bonito, Pedro Mil-Homens, Iván Peña-González, Maria Isabel Fragoso

**Affiliations:** 1 Faculty of Physical Culture Sciences, Autonomous University of Chihuahua, Chihuahua, Mexico; 2 University of Hull, Hull, UK; 3 Laboratory of Physiology and Biochemistry of Exercise, Faculdade de Motricidade Humana, CIPER, Universidade de Lisboa, Lisbon, Portugal; 4 Sports Research Centre, Department of Sport Sciences), Miguel Hernández University of Elche, Alicante, Spain; 5 Neuromuscular Research Lab, Faculdade de Motricidade Humana, CIPER, Universidade de Lisboa, Lisbon, Portugal

**Keywords:** Biological maturity, Soccer players, Skeletal age, Percentage of adult height, Biological techniques, Developmental biology

## Abstract

This study focus is to develop a new model to estimate skeletal age (SA) as a function of the state of biological maturation in male soccer players, and to propose cut-off points to classify the state of biological maturation based on the percentage of adult height (PAH). SA was determined in 747 Portuguese male soccer players, using the Tanner-Whitehouse (TW) 3 method, and PAH was predicted by TW3 (P-TW3) and Khamis-Roche (P-KR) methods. Subsequently, the sensitivity and specificity of the P-TW3 were estimated to classify late, on-time and early maturers to obtain cut-off points, by age; and to develop specific equations for each maturation stage. Both the model using P-TW3 and the model using P-KR showed a SA predictive capacity of 93%. The average differences were similar to zero. P-TW3 cutoff points were established by ROC curve analysis to identify late and early maturers according to their SA. Following, predictive models were developed to estimate SA according to maturity status. The predictive capacity of the models was 87.3% in late maturers, 92.3% in on-time maturers and 93.5% in early maturers. The prediction models are a reliable and cost-effective method to estimate SA in male soccer players.

## Introduction

Assessing the maturity status of male soccer players is crucial for talent identification, player development, and injury prevention strategies^1^. Considering maturity status in soccer is important to avoid maturity-related selection biases that may favor early-maturing players over late-maturing ones, prevalent across the globe^2–6^. These biases may result in the over-selection of early-maturing players, leading to long-term consequences for the development of late-maturing players, who often quit the sport prematurely due to a temporary physical disadvantage associated with the maturation process^2^. Grouping children by biological maturity rather than chronological age (a pratice known as bio-banding^1,7,8^) is commonly used in professional soccer academies ^1,3,9,10^. This approach helps coaches better assess players’ development^11^ and ensures that players of diferent maturity levels have equal opportunities to develop their skills and reach their full potential^8^.

Skeletal age (SA) is considered the gold standard for assessing biological maturation^12^, as it provides a direct measure of skeletal development^13^. SA is estimated by analyzing radiographs of specific bones in hand-wrist and comparing them to reference X-ray plates of different levels of skeletal maturation^14^. The degree of ossification, shape, and size of the bones are considered to determine the level of skeletal maturation using radiographs of the atlas system^15^. Two methods for determining SA have been commonly used: (1) the Tanner-Whitehouse (TW) method^14^ and (2) the Fels method^16^; this last provides maturity estimations for the radius and ulna, carpals and metacarpals, and phalanges^16^; while the TW method requires the use of a combination criterion that includes multiple bones of the hand and wrist, 13 bones that comprise the radius, ulna, and phalanges, while the 20 bones assessment incorporates the radius, ulna, phalanges, and carpals^14^.

The TW-3 method (TW3) is a more recent version of the TW method, based on European, South American, North American, and Japanese youth populations^14^. The TW3 method considers SA by assigning a maturation score to each bone and then combines these scores to determine an overall SA value^14^. Additionally, adult height can be predicted through the TW3 method. However, TW3 is a fairly complex, expensive, and time-consuming process that requires radiographic evaluation and subjective decision-making, which limits its utility in training environments; alternative non-invasive maturity estimation methods are commonly used within soccer environments^17^.

Non-invasive methods to estimate maturity status in soccer typically use somatic-based techniques^15^, where two indicators are commonly used: (1) Peak Height Velocity (PHV) which refers to the point during adolescence when an individual experiences the most rapid growth in height using the Fransen et al., Koziel et al., Mirwald et al., and Moore et al., methods^18–21^, and (2) Predicted adult height, using the Khamis & Roche method^22^, to estimate the percentage of adult height (PAH) observed at the time of measurement^15^.

Khamis & Roche’s^22^ method requires an accurate measurement of a child’s decimal age, standing stature, body mass (kg) and the accurate stature of both birth parents^22^. If the standing heights of both parents are available, then the mid-parent standing stature can be calculated in combination with the current stature and body mass of the child and used to estimate mature stature. This method uses smoothed values of the intercept and regression coefficients based on data from the Fels longitudinal study^16^. This method can be applied to healthy Caucasian children aged between 4.0 and 17.5 years. However, acknowledging error is crucial for the Khamis-Roche method^22^. Median error is slightly over 2 cm in boys and under 2 cm in girls (4.0-7.5 years)^7,23^. Accurate data collection reduces error to ~ 2.0 cm at the 50th percentile but can increase to ~ 0.3 cm at the 90th percentile for 11- to 15-year-olds^1,24^. This may lead to misclassification in bio-banding due to systematic errors rather than biological maturity^5^. Logistical challenges, often self-reported birth-parent height, contribute to errors. Validation recommends corrected self-reports or national mean stature values, though both may inflate errors. Despite Khamis Roche’s superior maturity estimation, precise composite anthropometric data are vital for effective player classification and physical development decisions^12^.

Although these methods have been applied to soccer players^9,24^, they were not designed for athletes at the beginning, which may be a factor to consider when using them. For example, Malina and Koziel^25^ observed in a longitudinal study conducted in Polish males that the Mirwald method^20^ tended to underestimate PHV at younger ages and overestimate it at older ages^25^. Parr et al.^24^ observed similar results in soccer players from the premier league football, where the estimated PHV by the Mirwald method^20^ at 13.0 years presented greater variation compared to the observed PHV obtained with longitudinal height records. The non-invasive methods offer advantages such as greater utility in large samples, less invasive, less time-consuming, do not require sophisticated equipment or highly qualified personnel and can be applicable to cross-sectional designs; however, they should be used with caution, as indicated by Fransen et al.^26^, due to its estimation errors, that may increase when applied to samples different from the original ones, from which non-invasive methods were designed and validated^14^. Despite these difficulties, sports sciences researchers should give special attention to the development of non-invasive methodologies that more accurately identify the timing and tempo of children and adolescents’ maturation^14^. Additionally, this will contribute to the bio-banding concept, recently applied to sports science, and aims to classify athletes considering their inter-individual differences in biological maturity status during adolescence. Bio-banding seeks to achieve fairer and more equitable grouping for competitions and talent identification processes by removing maturity selection bias^8^. Athletes aged 11 to 15 are commonly classified into bio-bands based on their percentage of predicted adult height (e.g., < 85% or > 90%) or their proximity to PHV, grouping them as pre-PHV, circa-PHV, or post-PHV. Developing equations to estimate skeletal age according to maturation status and establishing more specific cut-off points for the percentage of adult height can enhance the sensitivity and specificity of classifying soccer players based on their maturation status This approach can lead to better management of training loads, more balanced competition—which positively impacts the mental health of athletes—and a decreased injury risk, especially in late maturers. Therefore, the objectives of the present study were (1) to develop new equations to estimate skeletal age as a function of the state of biological maturation in male soccer players, and (2) to propose new cut-off points to classify the state of biological maturation based on the percentage of adult height in male soccer players.

## Methods

### Participants

The present non-experimental, prospective, cross-sectional and correlational study was approved by the Ethics Committee of Lisbon University, School of Human Kinetics (folio number CEIFMH: 3/2024), and implemented according to the Helsinki Declaration^27^. All participants assented to participate, and parents granted written informed consent.

A sample of 747 Portuguese male soccer players from one professional soccer academy, participated, aged 10 to 15, through convenience sampling. The sample was divided into two groups, group one for the development of the prediction model and group two for the validation of the model. In both groups, the sample size is considered sufficient according to Green et al.^28^, being *n* = 50 + 8 m, where m is the number of independent variables (PAH is the independent variable in the present study, represented by three other variables as weight, height and the average parental height, as will be described later in the methodology) so *n* = 74 minimum.

The inclusion criteria were: (a) belonging to a professional football academy for more than one year, (b) providing informed consent and assent; and the exclusion criteria were: (a) children with muscular, bone or joint injuries; (b) SA up ± 3 years of decimal age; (c) incomplete data (i.e. without parent’s height data).

Of the total sample, 570 soccer players were assigned to group 1 (G1), used to develop the SA predictive model; the remaining 177 players were assigned to group 2 (G2), the validation group, and who had their parents’ height, an important measure to estimate PAH (Table 1).

**Table 1:** Characteristics of chronological age, height, adult height by TW3, and parent´s height by group age in both design groups.

Age group	Age (years)				Height (cm)				Adult height TW3 (cm)				Parent´s height (cm)			
	*n*	Mean	±	SD	*n*	Mean	±	SD	*n*	Mean	±	SD	*n*	Mean	±	SD
Development equation group																
10 years	76	9.9	±	0.3	76	143.9	±	5.5	76	182.0	±	5.2				
11 years	119	11.0	±	0.3	119	151.0	±	6.6	119	183.4	±	5.7				
12 years	93	12.0	±	0.3	93	156.5	±	7.8	93	182.3	±	6.3				
13 years	120	12.9	±	0.3	120	162.7	±	8.0	120	182.0	±	6.2				
14 years	97	14.0	±	0.3	97	170.6	±	8.2	97	179.7	±	7.7				
15 years	65	14.9	±	0.3	65	173.8	±	7.9	65	179.5	±	7.4				
Validity equation group																
10 years	11	9.9	±	0.3	11	145.3	±	6.7	11	183.4	±	7.6	11	171.9	±	4.8
11 years	14	11.0	±	0.3	14	148.1	±	6.3	14	180.8	±	5.8	14	171.6	±	4.3
12 years	31	12.1	±	0.2	31	155.7	±	5.9	31	181.6	±	6.0	31	173.2	±	4.5
13 years	45	13.1	±	0.2	45	166.0	±	9.3	45	183.6	±	7.1	45	173.0	±	4.6
14 years	54	14.0	±	0.3	54	170.6	±	9.3	54	182.5	±	6.9	54	171.9	±	3.7
15 years	22	15.0	±	0.3	22	172.8	±	8.2	22	179.7	±	7.5	22	171.8	±	3.1

### Measures

Body mass (kg), stature (cm) and sitting height (cm) were measured following the International Society for the Advancement of Kinanthropometry (ISAK) protocol^29^. Leg length was calculated as the difference between stature and sitting height. Body mass was measured with a Secca body scale, model 761 7,019,009 to the nearest of 0.5 kg; and stature and sitting height were measured with a Siber-Hegner anthropometric kit to the nearest of 0.1 cm. All measurements were made by an ISAK anthropometric technician who holds a Level 2 qualification. The intra-observer technical error of measurements - %TEM (and coefficient of reliability - R) were well below the accepted maximum of 1% for stature (*R* ≥ 0.98) and 5% for skinfolds (0.90 ≤ *R* ≤ 0.98).

Skeletal age (SA). Biological maturity was assessed through SA evaluation according to the Tanner-Whithouse III method^14^ by two trained examiners. A left hand-wrist radiograph was taken using a portable X-ray model Ascor 110, which operates with a low level of radiation (set to 2 mA/s and 36 kV or 5 microsieverts). Subsequently, an experienced and qualified examiner (who has almost twenty years of experience using the TW method) assessed radiographs using a digital x-ray processor (Carestream Vitaflex CR) at the Faculty of Human Kinetics of Lisbon University. The mean difference of SA assessments was 0.03 years (± 0.04) and the inter-observer technical error measurement was 0.12 years (Table 2).

**Table 2:** Models summary for the prediction of skeletal age from the percentage of adult height by P-TW3 method in male soccer players by maturity status.

		B	Typical error	*p*-value	*r*	*R*²	Estimated error
All sample							
Intercept		-17.043	0.343	0.001	0.96	0.93	0.606
Coefficient P-TW3	0.336	0.004	0.001				
Late maturers							
Intercept		-18.445	0.976	0.001	0.93	0.87	0.599
Coefficient P-TW3	0.348	0.012	0.001				
On-time maturers							
Intercept		-15.742	0.476	0.001	0.96	0.92	0.541
Coefficient P-TW3	0.321	0.005	0.001				
Early maturers							
Intercept		-11.106	0.554	0.001	0.97	0.94	0.426
Coefficient P-TW3	0.275	0.006	0.001				

*P-TW3* Percentage of adult height by TW3 method.

Percentage of Adult Height. Three methodologies were used to estimate adult height: (1) the procedure developed by Tanner et al.^14^ (TW3) that uses the subject height in cm, and the scores obtained from an x-ray of the left wrist as variables; (2) the method established by Khamis and Roche (KR)^22^, considering the height in inches, weight in pounds, chronological age and the average of self-reported height of the parents in inches, as predictive variables; and (3) the Roche-Wainer-Thyssen (RWT)^30^ that uses the same variables as the KR procedure, but with different coefficients by sex and age. When adult height was estimated by the three methodologies, the percentage of adult height achieved at the time of observation was calculated for all players (P-TW3, P-KR and P-RWT).

### Procedure

Participants within G1 (*n* = 570; stature (cm): mean = 161.65; SD = 11.37; body-mass (kg/m^2^): mean = 54.95; SD: 12.93; Age (years): mean = 12.36, SD = 1.63) were used to develop the SA equation from P-TW3 as independent variable. This approach was administered to identify the coefficients used in the predictive equation, taking into account that SA and P-TW3 obtained through the same procedure, have a high correlation^14^; subsequently, P-TW3 was replaced by P-KR as a predictive variable, under the assumption that the P-TW3 and P-KR methods are not statistically different and possibly interchangeable as seen in the previously published analysis^31,32^. In the present study, differences between both methods were also contrasted.

Participants within G2 (*n* = 177; stature (cm): mean = 163.00; SD = 12.01; body-mass (kg/m^2^): mean = 50.6; SD: 12.4; Age (years): mean = 12.9, SD = 1.39) was considered to validate SA prediction equations with P-TW3 and P-KR. The P-RWT values were not used, as the P-RWT results, in this work, were statistically different from the P-TW3; conversely, P-KR predictions were similar to P-TW3 (see results section). Participants’ SA was used as a numerical and categorical variable classified into three different categories according to the difference between SA and chronological age late; on-time; and early maturer. On-time maturer was classified when the difference was within ± 1.0 years; late maturer when the difference was less than 1.0 years; and early maturer when the difference was over 1.0 year^33^.

Sensitivity and specificity of the P-TW3 were estimated to classify late, on-time and early maturers to obtain cut-off points, by age. Then soccer players were classified into late, on-time and early maturers to develop specific equations for each stage, in group 1. To validate the equations, players in group 2 were classified into late, on-time and early maturers based on the previously established cut-off points but using P-KR method instead.

### Statistical analysis

Kolmogorov-Smirnov test determined normality in SA measure and TW3 and KR methods, by sex. All variables showed a normal distribution (*p* > 0.200). A linear regression was used to estimate SA from the P-TW3 in group 1, and the assumptions of the linear regression model, such as linearity, normality, and homoscedasticity, were established. Simple regression analysis was performed to test linearity between the response variable (SA) and the predictor variable (percentage of adult height); residuals’ normality was determined using the Q-Q plot and the Kolmogorov-Smirnov test; homoscedasticity was seen through a scatter plot between the residuals and predicted values; and autocorrelation were analysed using the Durbi-Watson statistic.

To validate the SA prediction model in group 2 with SA obtained by TW3 and P-KR (1) Interclass correlation coefficient (ICC); (2) Bland-Altman graph; and (3) Kappa index, were used. Bland-Altman established the mean value of the differences and the average of both methods, and the Kappa index defined the level of concordance between SA categories using SA as the gold standard method. Means and standard deviations of observed SA, and SA predicted by P-TW3 and P-KR, plus P-TW3, P-KR and P-RWT were used and contrasted by ANOVA of repeated measures with Bonferroni pot-hoc test. To identify the cut-off for P-TW3 and classify late, on-time and early maturers a receiver operating characteristic (ROC) curve analysis was applied. Kappa index and a Spearman correlation coefficient were used to identify the concordance between the maturity stages (late, on-time and early) by SA observed and the new cut-off from P-TW3. All statistical tests were performed at a 95% confidence level.

## Results

### Group 1: model development (general equation)

The linear regression model to estimate SA from P-TW3 presented a predictive capacity of 92.9% and a correlation coefficient of 0.96 (*p* ≤ 0.001). The equation to estimate SA was SA= -17.043 + (0.336 * P-TW3).

Linearity was verified through the relationship between P-TW3 as the predictor variable and SA as the response variable, observing a correlation coefficient of 0.96 (*p* ≤ 0.001). Homoscedasticity was verified through the constant variance of the residuals, where a linear regression analysis was used considering the predicted values of SA as an independent variable and the residuals as the dependent variable; there was not any relationship (*r* ≤ 0. 001, R^2^ ≤ 0.001, BO = -2.18^− 15^, B1 = 0.000, *p* > 0.999). To examine autocorrelation, the Durbin-Watson statistic was employed yielding a value of 1.96, which indicated that the residuals were indeed independent. Lastly, the normality of the residuals was assessed using a Q-Q plot and histogram; both of them displayed a normal distribution.

### Group 2: model validation

Table 3 presents the characteristics of SA and the percentage of adult height by the three methods. No differences were found between P-TW3, P-KR and P-RWT through ANOVA of repeated measures with Bonferroni post-hoc test at all ages (*p* = 0.066 to 0.601). However, although not significant, p-values closer to 0.05 were found between P-RWT and P-TW3, so P-KR was used hereafter.

**Table 3:** Descriptive data of skeletal age and percentage of adult height by different methods in male soccer players.

Age (years)	*n*	SA observed (years)			SA *P*-TW3 (years)			SA *P*-KR (years)			*P*-TW3 (%)			*P*-KR (%)			*P*-RWT (%)		
		Mean	±	SD	Mean	±	SD	Mean	±	SD	Mean	±	SD	Mean	±	SD	Mean	±	SD
10	11	9.3	±	1.3	9.6	±	0.2	9.4	±	0.3	79.2	±	0.7	78.7	±	1.0	78.7	±	0.9
11	14	10.8	±	1.2	10.5	±	0.5	10.3	±	0.5	81.9	±	1.5	81.3	±	1.5	81.5	±	1.3
12	31	11.9	±	1.1	11.8	±	0.6	11.7	±	0.7	85.8	±	1.7	85.6	±	2.0	86.0	±	2.1
13	45	13.3	±	1.0	13.3	±	0.8	13.2	±	0.9	90.4	±	2.5	90.0	±	2.7	89.8	±	2.2
14	54	14.1	±	0.9	14.3	±	0.9	14.3	±	0.9	93.4	±	2.8	93.2	±	2.7	93.2	±	2.3
15	22	15.1	±	0.9	15.3	±	1.0	15.2	±	0.7	96.1	±	3.0	95.8	±	2.1	96.0	±	1.8

No differences were found in Skeletal age and Percentage of adult height among the different methods, only at 14 years between SA observed and SA predicted by P-TW3 (*p* = 0.002).

*SA* Skeletal age; *P-TW3* Percentage of adult height by Tanner-Whitehouse III method; *P-KR* Percentage of adult height by Khamis and Roche method; *P-RWT* Percentage of adult height by Roche-Wainer-Thissen method; *SD* Standard deviation.

In addition, high correlations were found between the three methodologies, P-TW3 and P-KR of 0.94 (*p* ≤ 0.001) and P-TW3 and P-RWT of 0.93 (*p* ≤ 0.001).

**Fig. 1:**
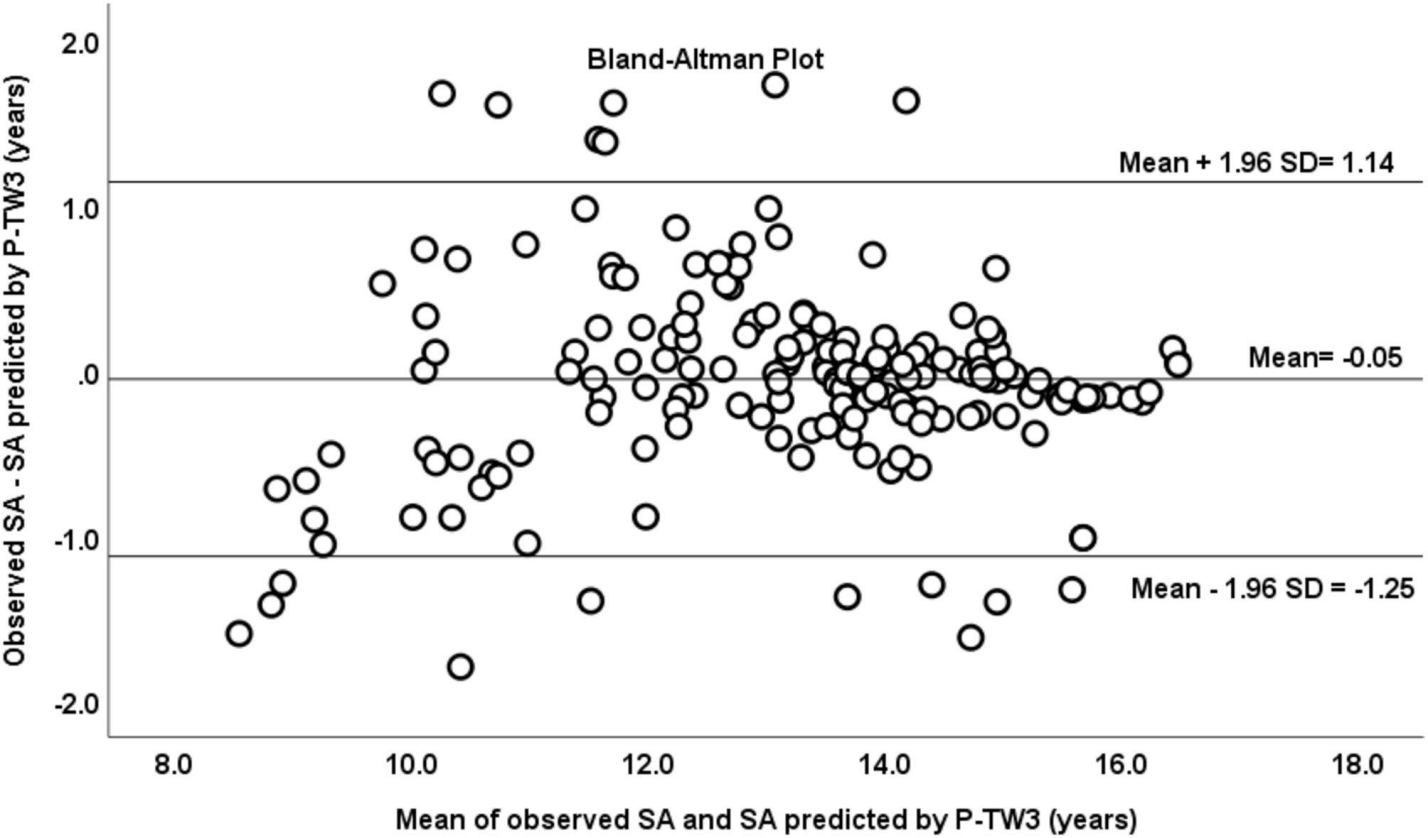
Bland–Altman plot of SA observed and SA predicted by P-TW3 method in the model validation group.

The reliability analysis between observed SA and SA predicted by P-TW3 showed a correlation coefficient of 0.94 (*p* < 0.001), and an ICC of 0.97 (*p* < 0.01) were found, considered as a very strong level of agreement or consistency. Bland-Altman analysis (Fig. 1) showed an average of the differences between observed SA and estimated SA of -0.053 ± 0.061 years, being similar to zero (*p* = 0.250, CI -0.143 to 0.037); and 91% of the data were within the limits of agreement (CI = 1.14 to -1.25). Likewise, no trend was found between the differences obtained by both methods and the average of SA between both methods, analyzed through linear regression (*r* = 0.034, R^2^ = 0.001, *p* = 0.649). On the other hand, a Kappa index of 0.58 (*p* < 0.001) which is considered as weak agreement, a Kendall’s tau-b correlation coefficient of 0.66 (moderate agreement; *p* < 0.001) and a percentage of agreement of 81% (subjects classified similarly between both methods) were found between the three maturation status categories obtained from the observed SA and the SA predicted by the new model. Subsequently, the variable P-TW3 was substituted in the equation by P-KR, finding an ICC of 0.93 (Very good agreement); *p* < 0.001) and a correlation coefficient of 0.86 (good agreement); *p* < 0.001) between the observed SA and the SA predicted by P-KR.

**Fig. 2:**
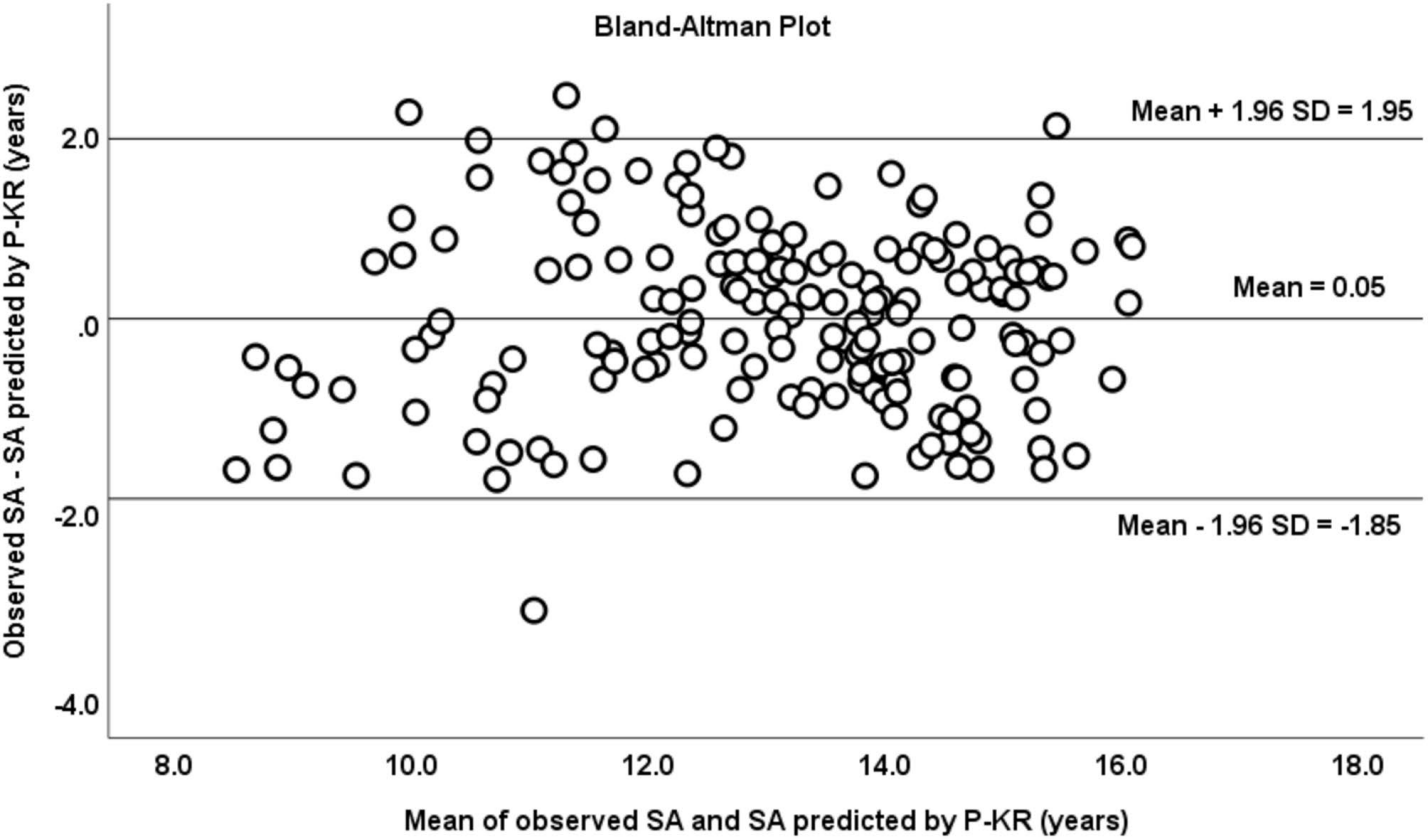
Bland–Altman plot of SA observed and SA predicted by P-KR method in the model validation group.

Bland-Altman analysis (Fig. 2), the mean differences were similar to zero (x̄= 0.050 ± 0.972, CI -0.094 to 0.194, *p* = 0.493) and the 97% of the data were within the limits of agreement (CI = -1.85 to 1.95). A linear regression analysis demonstrated no significant relationship between the differences and the average of SA obtained by both methods (*r* = 0.010, R² < 0.001, *p* = 0.897). Reliability by the estimated biological maturation categories presented a Kapa index of 0.123 (*p* = 0.022) (there is a weak agreement between the two classification methods) and the Kendal’s Tau-b correlation coefficient was 0.276 (*p* < 0.001), with 63% of coincidence in the classification.

### The cut-off for the percentage of adult height

P-TW3 cut-off points were analyzed through a ROC curve analysis to identify late and early maturers classified based on their SA. Table 4 shows the percentage of adult height corresponding to early, late, and on-time maturation. For instance, to classify a 13-year-old adolescent as an on-time maturer, their percentage of adult height should fall within the range of > 86.7 to < 91.3. The observed area under the ROC curve (AUC) was relatively low in the 10-year-old group, with values of 0.71 for identifying late maturers and 0.82 for early maturers. Conversely, in the remaining age groups, the AUC was high, above 0.90, except for the 12-year-old group, where an AUC of 0.87 was found for identifying late maturers. On the other hand, the classification of maturation status based on percentage of adult height showed an adequate degree of agreement with the classification by SA, mainly for the age groups between 11 and 15 years, where the Kappa index ranged from 0.52 to 0.76 (*p* < 0.05) and a Spearman correlation coefficient between 0.69 and 0.91 (*p* < 0.05).

**Table 4:** Cut-off values to identify late, on-time and early matures from PAH and concordance test between PAH and SA maturity status classifications in male soccer players.

Age (years)	*n*	Mean	SD	Late	Early							% agreement	Kappa	Spearman
				Inferior	AUC (CI)	SE	SP	Superior	AUC (CI)	SE	SE			
10	76	79.1	1.0	78.3	0.71 (0.59, 83)	80%	45%	80.6	0.82 (0.51, 1.00)	75%	94%	64%	0.267	0.381
11	119	82.3	1.9	81.1	0.90 (0.84, 0.95)	87%	72%	84.3	0.97 (0.95, 0.99)	86%	94%	78%	0.632	0.744
12	93	85.8	2.7	84.5	0.87 (0.79, 0.95)	84%	76%	87.1	0.91 (0.85, 0.97)	82%	82%	68%	0.518	0.692
13	120	89.4	2.8	86.7	0.97 (94, 1.00)	95%	89%	91.3	0.94 (0.89, 0.98)	76%	93%	83%	0.69	0.774
14	97	95.0	3.5	91.1	0.91 (81, 1.00)	87%	80%	96.6	0.99 (0.98, 1.00)	95%	98%	86%	0.756	0.91
15	65	96.8	3.0	93.8	0.98 (0.95, 1.00)	91%	100%	99.0	0.96 (91, 1.00)	85%	93%	83%	0.713	0.814
10 a 15												78%	0.629	0.751
11 a 15												80%	0.668	0.78

SA = Skeletal age; PAH = Percentage of adult height; SD = Standard deviation; AUC = Area under the curve; SE = Sensibility; SP = Specificity.

### Equations for maturity status (specific equations)

After the biological maturity status classification of soccer players, based on the percentage of adult height, a predictive model was developed to estimate SA derived from P-TW3 measurement. The predictive capacity of the models was 87.3% in late maturers (*r* = 0.93, *p* < 0.001), 92.3% in on-time maturers (*r* = 0.96, *p* < 0.001) and 93.5% in early maturers (*r* = 0.97, *p* < 0.001). Table 2 presents the summary of the models and the equations are presented below:

$${\mathbf{Late}}{\text{ }}{\mathbf{maturing}}\;{\text{SA}}={\text{ }} - {\text{18}}.{\text{445 }}+{\text{ }}\left( {0.{\text{348}}*{\text{P}} - {\text{TW3}}} \right).$$

$${\mathbf{On}} - {\mathbf{time}}{\text{ }}{\mathbf{maturing}}:{\text{ SA}}={\text{ }} - {\text{15}}.{\text{742 }}+{\text{ }}\left( {0.{\text{321}}*{\text{P}} - {\text{TW3}}} \right).$$

$${\mathbf{Early}}{\text{ }}{\mathbf{maturing}}:{\text{ SA}}={\text{ }} - {\text{11}}.{\text{1}}0{\text{6 }}+{\text{ }}\left( {0.{\text{275}}*{\text{P}} - {\text{TW3}}} \right).$$

Linear regression assumptions for the three models were fulfilled, normality of residuals was confirmed; there was an adequate linearity between observed SA and PTW-3; no autocorrelation was found based on the Durbin-Watson statistic, which was 2.0 for late, 1.6 for on-time and 1.4 for early; and homoscedasticity was tested through the constant variance of the residuals, using a linear regression analysis between SA predicted and the residuals; no relationship was revealed in the three groups (*r* < 0.001, R^2^ < 0.001, *p* = 1.000).

### Validation of the equations by maturity status

**Fig. 3:**
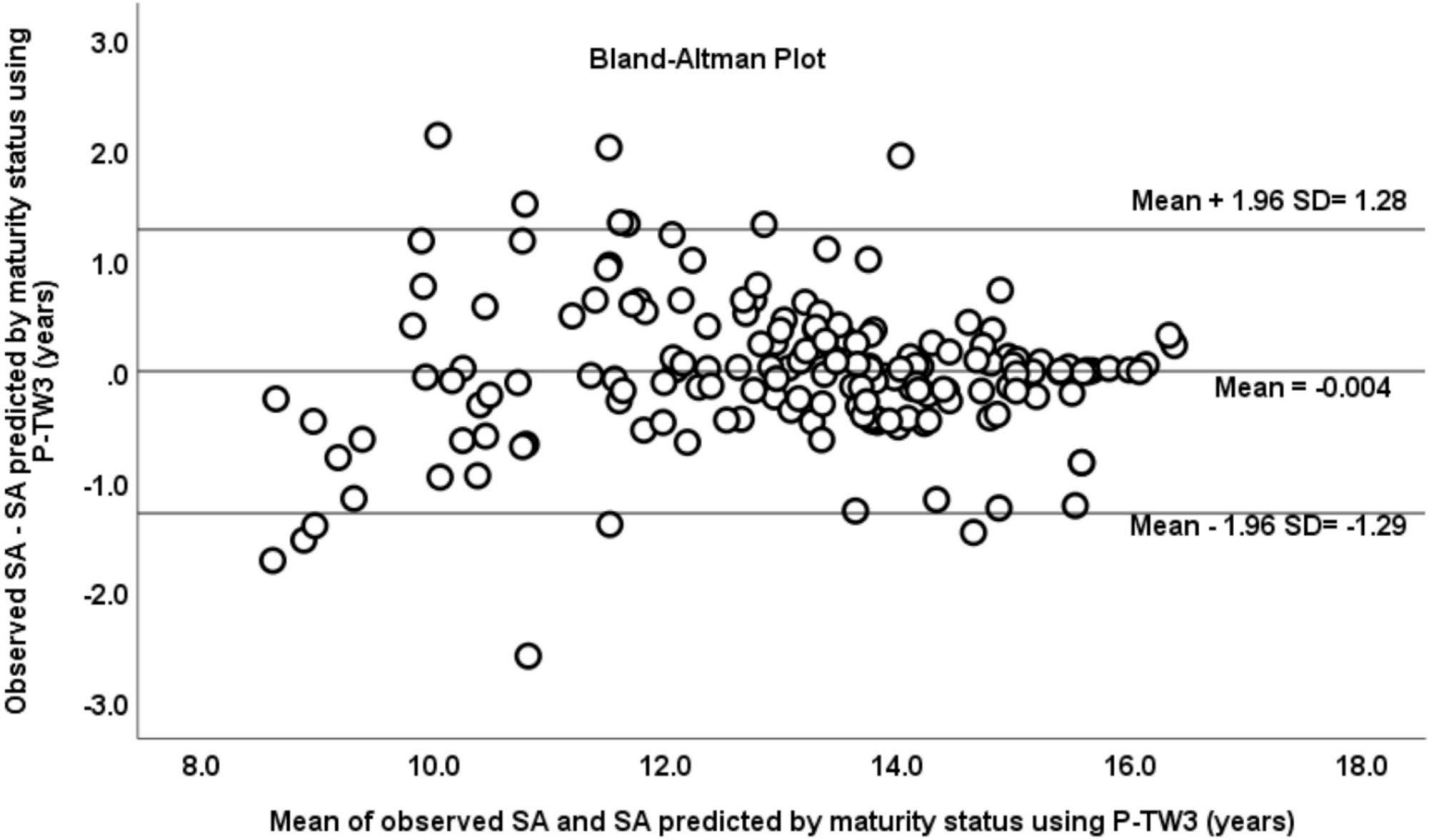
Bland–Altman plot of SA observed and SA predicted by P-TW3 method, using specific equations by maturity status.

**Fig. 4:**
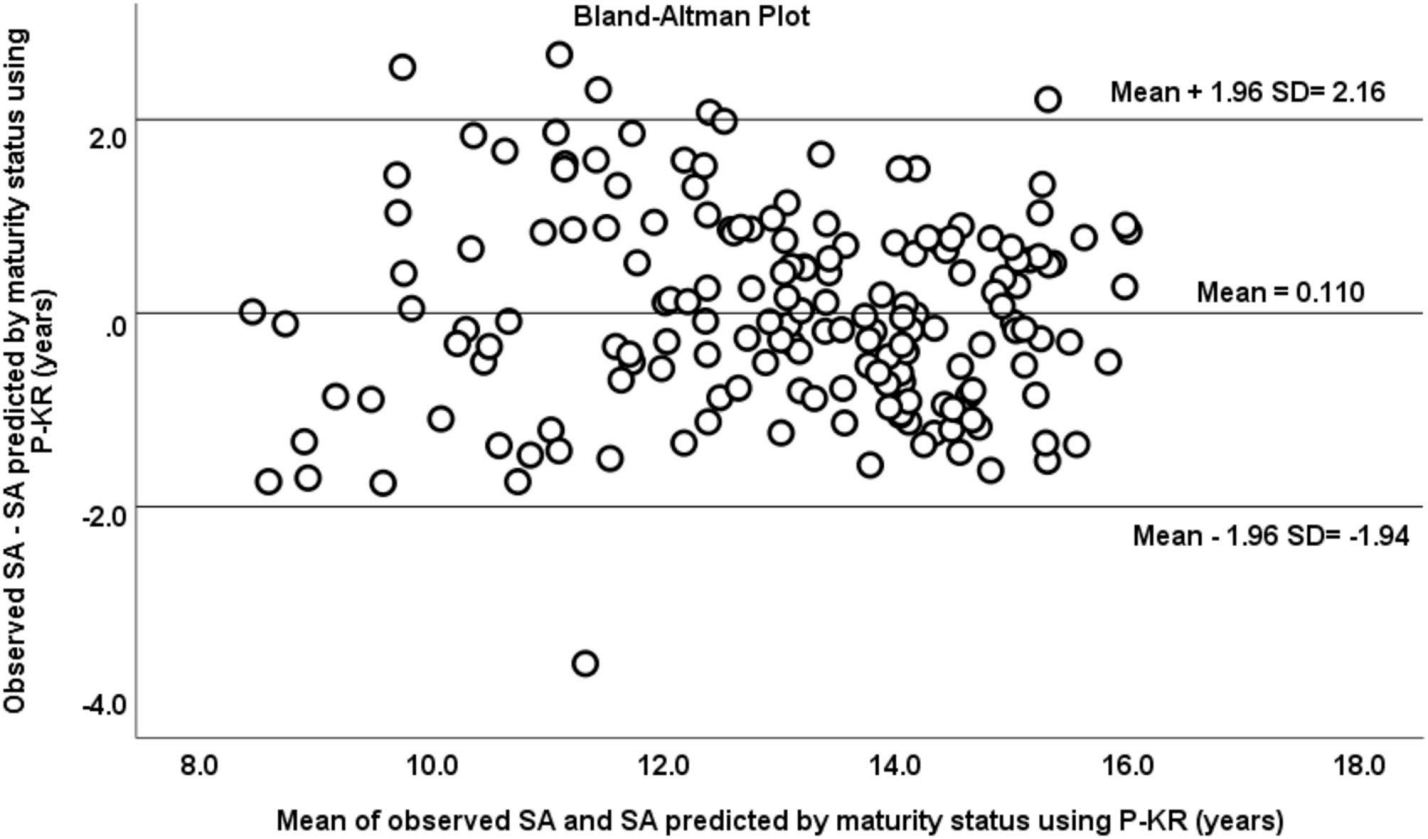
Bland–Altman plot of SA observed and SA predicted by P-KR method, using specific equations by maturity status.

The specific equations for each biological maturation stage were applied using P-TW3 as a predictor variable. It was observed a correlation coefficient and ICC of 0.937 and 0.967, respectively (*p* < 0.001) between observed and predicted SA. The mean of differences between observed and predicted SA was similar to zero (x̄ = -0.004 ± 0.656, CI -0.107 to 0.093, *p* = 0.930), and no trend was observed between the differences and the average of observed and predicted SA (*r* = 0.027, R^2^ < 0.001, *p* = 0.722). Based on the Bland-Altman plot, it was observed that 93% of the data were within the limits of agreement (CI = -1.29 to 1.28) (Fig. 3). When P-TW3 was substituted for P-KR in the maturity equations, the observed ICC was 0.91 and the correlation coefficient was 0.84 between the observed and predicted SA (Fig. 4). The mean of differences between both methods was similar to zero (x̄ = -0.110 ± 1.047, CI -0.045 to 0.266, *p* = 0.162). No relationship was found between the differences and the average of SA by both methods (*r* = 0.026, R^2^ < 0.001, *p* = 0.730). The 97% of the data were within the agreement limits based on the Bland-Altman plot (CI -1.94 to 2.16).

## Discussion

The present study is the first study to propose a non-invasive method for estimating SA in soccer players and to estimate maturity status from the percentage of adult height via the use of the P-KR method. Therefore, the present study aimed to establish the coefficients of the model used to estimate SA through the P-TW3 method, which presents good reliability in the absence of the true gold standard, for the assessment of SA^34^. The main findings of this study are three-fold: (1) the model predicted SA from P-TW3 presents a R^2^ of 0.93 and a correlation coefficient of 0.96 (*p* < 0.001). Subsequently, the variable P-TW3 was substituted in the equation by P-KR, finding an ICC of 0.93 (*p* < 0.001) and a correlation coefficient of 0.86 (*p* < 0.001) between the SA observed and the SA predicted by P-KR; (2). Cut-off values were proposed for the percentage of adult height to identify late, on-time and early maturers, with adequate sensitivity and specificity; (3). Specific equations were developed to estimate SA for maturity status, the predictive capacity of the models was 87.3% in late maturers (*p* < 0.001), 92.3% in on-time maturers (*p* < 0.001) and 93.5% in early maturers (*p* < 0.001).

In previous research, a similar equation has been developed to estimate SA from P-KR within the general Portuguese population^32^. This equation presented a good predicting capability, 84% in girls and 83% in boys when P-KR was used in Flores et al.^31^. Flores et al.^32^ established this by implementing a two phased methodological approach comprised of initially, establishing model coefficient determined the P-TW3 method, which uses SA from the Tanner et al., III method^14^, reducing prediction errors. Then subsequently, substituting the P-TW3 value with the P-KR value, assuming that both protocols estimate a similar percentage of adult height. It has been demonstrated that the percentage of adult height using the P-KR and P-TW3 methods for predicting SA can be used interchangeably, as no significant (*p* > 0.1) difference in non-athlete boys and girls across all age groups in previous works was present^33,34^, and as observed in this study (*p* = 0.099 to 0.601).

To our knowledge, no skeletal age estimate method has been previously proposed in the athlete population, making the current work the first to introduce such an approach. Nevertheless, like all other methods developed to assess biological maturation status, it should be taken with caution. Although the general equation showed a very good (93%) predictive capability for SA, the reliability to estimate different maturation categories (late, on-time, and early) was low (Kappa index of 0.58); for that reason, the present study proposes cut-off points for the percentage of adult height, which showed an adequate degree of agreement with the classification by SA, between 11 and 15 years. The cut-off points have good validity in classifying the state of biological maturation based on the percentage of adult height in young male soccer players. Indeed, a recent systematic review observed low agreement between different non-invasive methods for classifying maturation status based on PAH, SA, or PHV, similar to the degree of agreement observed for the equation proposed in the present study. However, the approach proposed in this study, using cut-off points for PAH, may help in the classification of biological maturation status^35^.

Recent studies found that PHV in North American boys and girls occurs between 85% and 96% of adult height, with an average of approximately 90%, this range of the percentage of adult height has been used, in professional soccer academies and, to classify maturity status^8,17,36^ and for maturity-specific training^6,9,26^. The maturity bio-bands, for males, based on cumulative growth and percentage of adult height, classify the subjects as pre-pubertal when their percentage of predicted adult height is < 85% (Pubic Hair - PH1), early pubertal between 85 and 90% (PH2), at the growth spurt when their percentage of adult height is between 85% and 96% (PH2, PH3 and PH4), and as post-pubertal when they show a percentage of adult height > 96%^7^. Similar criteria have been used by other investigators^37^, classifying soccer players aged age 13.3 ± 1.5yrs as pubertal (88–94%), or post-pubertal (> 95%). Therefore, based on the percentages of adult height by chronological age presented in this study, athletes can be grouped; for example, a 13-year-old soccer player with a percentage adult height lower than 86.7% can train with the 12-year-old group of athletes; likewise, a 13-year-old athlete with an adult height% higher than 91.3% can receive training loads similar to the 14-year-old group of athletes. The cutoff points for the percentage of predicted adult height in the present study not only facilitate the identification of an athlete’s pubertal status but also enable classification of the athlete’s maturational status as late, on-time, or early. This allows for the reassignment of players to groups with similar maturity levels within a chronological age group or to a more or less advanced age group^1^.

In addition, the cutoff points for the percentage of adult height, presented in this study, showed a good degree of agreement with the late, on-time, and early maturers classification obtained through the SA. Malina et al.^13^, observed a 63% degree of agreement between skeletal age maturity status and z-scores transformation of the percentage of adult height results, based on the means and standard deviations presented in the Berkeley Guidance Study^38^, a lower degree of agreement respect to the present study (68 to 86%) compared to this work. The current approach to identifying maturity status aligns with recent methods involving PHV. In this approach, individuals achieving PHV between 12.2 and 13.6 years are categorized as average maturers, while those reaching it before 12.2 years are labelled as early maturers, and those after 13.6 years are designated as late maturers^39^. Once the cut-points were established using the percentage of adult stature in the present study, all the players were classified as early, late and on-time maturers based on the percentage of adult height, specific equations for late, on-time and early maturers to predict SA, from P-KR, were generated. Therefore, the suggestion is the following: (1) first classify the player based on his maturation status using the percentage of adult height; and subsequently, estimate the SA by using the specific equation; (2) if the purpose is the use of SA only, then a general equation may be used.

The use of SA as a measure of biological maturation is important in talent identification and selection processes in soccer, as it can provide valuable information about the potential for future growth and development. Ostojic et al.^40^ observed that late maturers from the cohort of successful 14-year-old soccer players, with a biological age delay, of more than 6 months, are more likely to achieve success in the top-level game (professional debut), compared with their early-maturing counterparts. Furthermore, maturity status could help in designing training programs that cater to their specific needs and skills, which in turn can improve their performance and reduce the likelihood of injuries. According to a cohort study, early maturers are more prone to injuries, while those classified as mature (with fully ossified hand and wrist bones) have the lowest risk of experiencing lower extremity injuries^41^.

Biological maturation varies significantly among individuals of the same chronological age, impacting physical performance, injury risk, and the effectiveness of biological adaptations in football. Accurate assessment of biological maturation status allows coaches and health professionals to design training programs that are tailored to the individual needs of athletes (Towlson et al., 2020). The present study aims to address this issue by providing a methodology to identify maturity status. Adjusting training loads based on maturation is essential to optimize performance and minimize the risk of injury. For instance, athletes experiencing growth spurts may need modifications in training intensity and volume to take advantage of their training window, while late maturers might require adjustments to prevent overload injuries^42,43^. Considering biological maturation in training plans can also have positive psychological effects by preventing unfair comparisons with peers at different maturation stages^17^.

The non-invasive equations proposed in the present study complement other non-invasive methods described in the literature for estimating the biological maturation status, such as the ultrasound method for estimating skeletal age^44^. Ultrasound imaging has emerged as an important non-invasive technique because it does not expose individuals to ionizing radiation, making it a safer option, especially for young athletes. Moreover, it has shown a good correlation with X-ray-derived bone ages, suggesting that ultrasound could be a viable alternative for regular use in sports setting^45^. However, ultrasound imaging still requires expensive equipment and qualified personnel compared to anthropometry, which is more accessible and easier to measure. These two methods (skeletal age estimation through ultrasound and the use of anthropometry-based equations) provide an alternative for estimating the tempo of biological maturation and complement other non-invasive methods already proposed for estimating the timing of biological maturation, such as the assessment of PHV.

Finally, some limitations should be considered. Firstly, the study only included male soccer players, secondly, the majority of the sample portrays a professional soccer reality of a specific academy in Portugal, which may be very good as a specific solution but a limitation if we want to generalize the findings to other populations, which may present different somatic and growth rates. Thirdly, the Tanner et al.^14^ III method was used as a criterion to measure SA, although the SA is considered the gold standard method for estimating biological maturation^13^ and its use and application in sports science is widely justified^1,37^; There is currently no consensus on the most accurate method for determining skeletal age. This is due to a number of factors, such as the specific bones and criteria used to assess skeletal maturity, the rationale behind assigning skeletal age, the choice of radiograph to evaluate (hand/wrist, knee, or ankle), differences in the growth patterns of adolescents from different ethnicities used in the reference samples for validating the methods, and the diversity of the populations used to derive skeletal age estimates, which could explain some of the differences between various methods^35^. Fourth, a significant limitation of our study is its exclusive focus on male participants, which restricts the applicability of our findings to female athletes. This represents a considerable knowledge gap that should be addressed in future research. The study was conducted only on males due to the convenience of obtaining a larger sample size in the youth academy, where there is a higher number of male players.

Based on these findings, it can be concluded that the prediction models developed in this research can be used as a reliable and cost-effective method to estimate SA in male soccer players. The proposed cut-off points based on the percentage of adult height can also be used to classify the state of biological maturation in male soccer players. The use of non-invasive methods, such as estimating the percentage of adult height and SA, can provide valuable information about the potential growth and development in soccer players. However, further research is needed to validate these prediction models, particularly through longitudinal studies that assess their performance and cut-off points over extended periods. This approach will help determine their accuracy and reliability in tracking individual maturity trajectories among male soccer players, and aid in refining these models to enhance their applicability across various sports contexts.

## Data Availability

Authors will make available upon request, to editors and reviewers, any previously unreported data, custom computer code or algorithm used to generate results that are reported in the paper and central to its main claims. If someone wants to request the data from this study please contact the corresponding author.
